# Antarctic Crabs: Invasion or Endurance?

**DOI:** 10.1371/journal.pone.0066981

**Published:** 2013-07-03

**Authors:** Huw J. Griffiths, Rowan J. Whittle, Stephen J. Roberts, Mark Belchier, Katrin Linse

**Affiliations:** British Antarctic Survey, Cambridge, United Kingdom; Université du Québec à Rimouski, Canada

## Abstract

Recent scientific interest following the “discovery” of lithodid crabs around Antarctica has centred on a hypothesis that these crabs might be poised to invade the Antarctic shelf if the recent warming trend continues, potentially decimating its native fauna. This “invasion hypothesis” suggests that decapod crabs were driven out of Antarctica 40**–**15 million years ago and are only now returning as “warm” enough habitats become available. The hypothesis is based on a geographically and spatially poor fossil record of a different group of crabs (Brachyura), and examination of relatively few Recent lithodid samples from the Antarctic slope. In this paper, we examine the existing lithodid fossil record and present the distribution and biogeographic patterns derived from over 16,000 records of Recent Southern Hemisphere crabs and lobsters. Globally, the lithodid fossil record consists of only two known specimens, neither of which comes from the Antarctic. Recent records show that 22 species of crabs and lobsters have been reported from the Southern Ocean**,** with 12 species found south of 60°S. All are restricted to waters warmer than 0°C, with their Antarctic distribution limited to the areas of seafloor dominated by Circumpolar Deep Water (CDW). Currently, CDW extends further and shallower onto the West Antarctic shelf than the known distribution ranges of most lithodid species examined. Geological evidence suggests that West Antarctic shelf could have been available for colonisation during the last 9,000 years. Distribution patterns, species richness**,** and levels of endemism all suggest that, rather than becoming extinct and recently re-invading from outside Antarctica, the lithodid crabs have likely persisted, and even radiated, on or near to Antarctic slope. We conclude there is no evidence for a modern-day “crab invasion”. We recommend a repeated targeted lithodid sampling program along the West Antarctic shelf to fully test the validity of the “invasion hypothesis”.

## Introduction

In recent years, the ‘crab invasion’ story has become a metaphor for climate change in the Antarctic marine realm, both in scientific literature and the media. The underlying premise of the ‘invasion hypothesis’ is that crabs were driven out of Antarctica between 40 and 15 million years ago and are now returning as similarly ‘warm’ and favourable habitats become available once more. This hypothesis remains untested, however, and does not include evidence derived from repeated sampling showing a change or expansion in crab ranges. The aim of this paper is, therefore, to examine, in detail, the past and present geographic and bathymetric distribution records of crabs and lobsters from the Southern Ocean (SO) to determine the nature of their historical and Recent (i.e., all living specimens) presence in the region.

Parts of Antarctica are among the fastest warming areas on the planet. The rate of atmospheric temperature increase on the Antarctic Peninsula is increasing at several times the global mean leading to widespread retreat of glaciers on the Antarctic Peninsula [1]–[3]. Increases in ocean surface temperatures have been detected to the west of the Antarctic Peninsula [4]. Although small increases, they are possibly significant as many believe that the Antarctic marine fauna is particularly sensitive to changes in temperature [5]–[8]. Furthermore, recent increases in air temperatures coupled with ever-increasing human activity have allowed the recorded introduction of many non-native species of terrestrial plants and animals in parts of Antarctica and the sub-Antarctic [9].

While invasive species in the terrestrial realm are well-documented, evidence of similar invasions in the Antarctic marine environment remains sparse [9]. Much of this uncertainty stems from a lack of baseline data in Antarctica to compare new distribution records against. Over recent years, great efforts have been made to fill this vital gap in knowledge by accumulating all published information into online biogeographic data portals [10]. The Marine Biodiversity Information Network of the Scientific Committee on Antarctic Research (SCAR-MarBIN) [11] represents the largest single collection of georeferenced Antarctic marine biodiversity data ever compiled, from over 270,000 locations south of the Polar Front (PF) [10]. Over 70% of all benthic records come from the continental shelf, highlighting the paucity of records from the slope and deep sea [12]. In recent decades scientific attention has moved towards the deeper regions of the SO and has largely focussed on the Weddell Sea [13].

**Figure 1:**
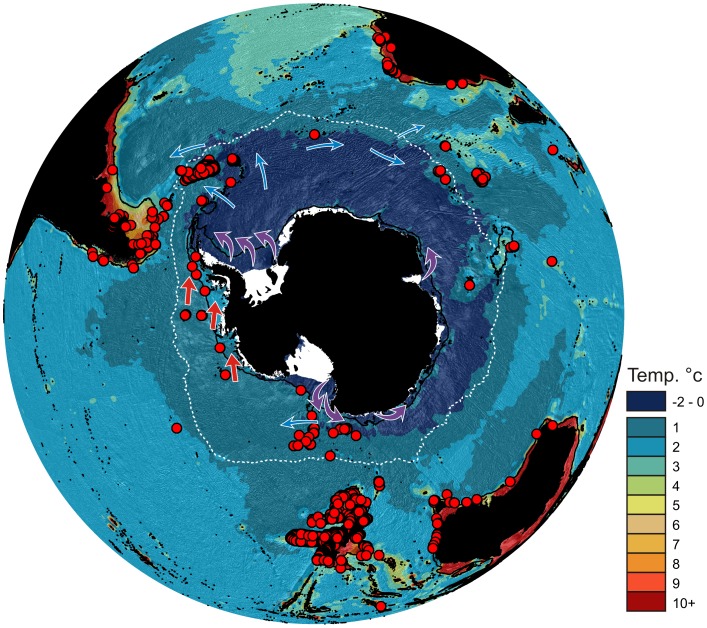
Geographic distribution of recorded Lithodidae from the Southern Ocean with seafloor temperature. Lithodid crab records = red circles, mean position of Polar Front (Moore et al., 1999) = white dotted line, Purple arrows = ABW production, Blue arrows = ABW direction (Orsi 2010 Nat Geo Sci), Red arrows = Circumpolar Deep Water on shelf. Temperature data from Clarke *et al.,* 2009.

During recent years there has been much speculation and interest following the discovery of lithodid crabs in the deeper water around Antarctica (Fig. 1), centred on the hypothesis that these crabs might be poised to invade the Antarctic shelf with warming seas, potentially decimating its native fauna [14]–[16].

**Figure 2:**
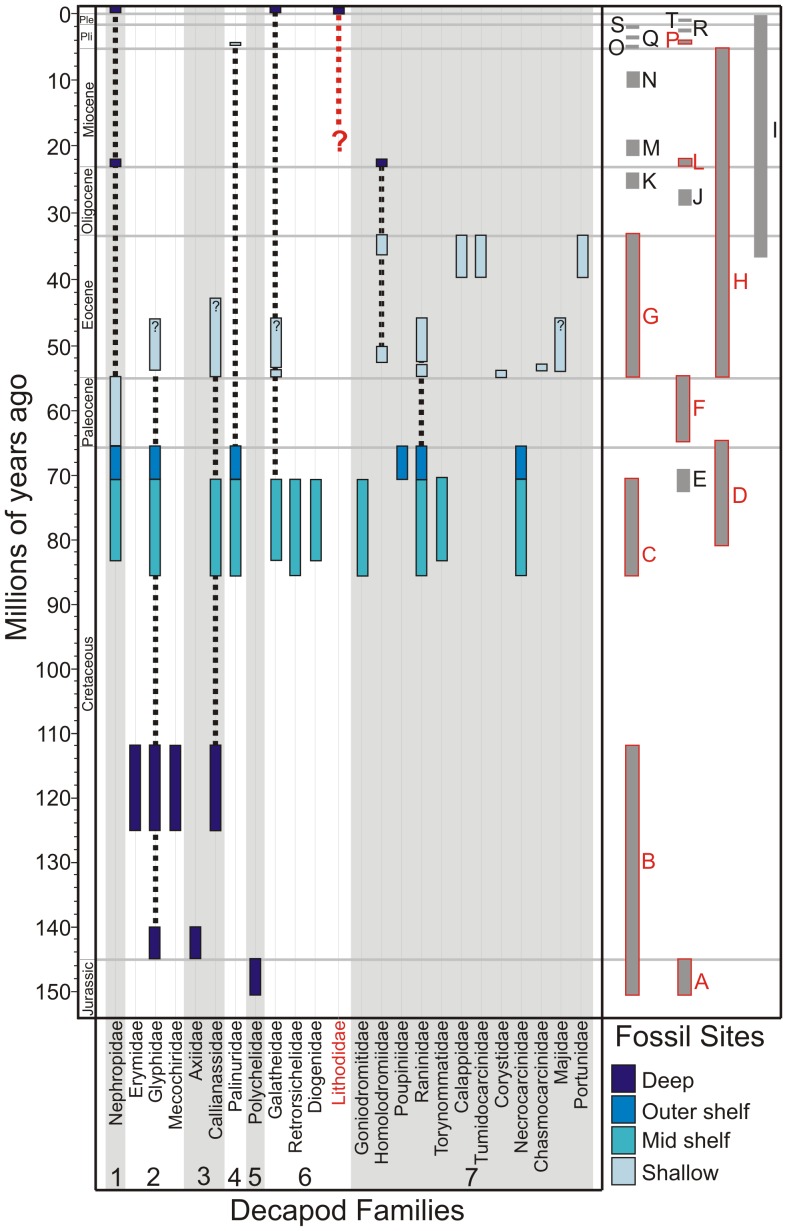
Ranges of fossil decapods in Antarctica. Bars represent family ranges, colours show water depth of depositional unit in which the fossils were found. Dotted lines are inferred ranges between occurrences. ? = inferred position for end of a range. Red dashed line indicates the uncertainty of the fossil history of the Lithodidae due to the complete lack of a fossil record. All known marine macrofossil sites are shown on the right hand side (grey bars). Red letters indicate the sites in which crab fossils were found. References for ranges, unit water depths and fossil sites are given in Appendix S1. Infraorders labelled as; 1 = Astacidea, 2 = Glypheidea, 3 = Axiidea, 4 = Achelata, 5 = Polychelida, 6 = Anomura & 7 = Brachyura. Stratigraphic units labelled as; Nordenskjöld Formation = A, Fossil Bluff Formation = B, Santa Marta Formation = C, López de Bertodano Formation = D, Snow Hill Island Formation = E, Sobral Formation = F, La Meseta Formation = G, McMurdo Sound erratics = H, DSDP drill holes = I, Polonez Cove Formation = J, Destruction Bay Formation = K, Cape Melville Formation = L, Battye Glacier Formation = M, Hobbs glacier Formation = N, Prospect Mesa gravels = O, Bull Pass Formation = Q, Scallop Hill Formation = R, Cockburn Island Formation = S and Weddell Sea Formation = T, Sørsdal Formation = P.

The assumptions upon which the ‘invasion hypothesis’ are based are potentially flawed, primarily because they rely on a geographically and spatially poor fossil record for Antarctic decapods with many significant temporal gaps and few ice-free fossiliforous rock outcrops [17]. The fossil record of decapods in Antarctica ranges from the Jurassic [17] (Fig. 2). Seven different infraorders have been identified, but, overall, the fossil record of decapods does not reflect the distribution pattern of Recent decapods in Antarctica. The global fossil history of Lithodidae is extremely poor, consisting of only two occurrences, one from the Middle Miocene of New Zealand and a broken claw from the Miocene of Japan [18]–[19]. This may indicate a very low preservation potential for the group as a whole, or a preference for deep water habitats [18]. Lithodid fossils are completely absent from the Antarctic fossil record (Fig. 2), but Lithodid crabs are common in modern Antarctic seas. Conversely, brachyuran crabs are virtually absent from present day high-Antarctic shelves [20], but the Brachyura has the greatest diversity of any infraorder within the Antarctic fossil record (Fig. 2), with specimens found at several localities in units that were deposited in all water depths, from shallow to deep.

The earliest records of Recent and modern day SO crab and lobster species date back to the Challenger expedition with records of *Lithodes murrayi* and *Paralomis aculeata* from the Prince Edward Islands in 1873 [21]. The first record of a crab in Antarctica (south of 60°S) is of a single record of the brachyuran crab *Halicarcinus planatus* from shores of the South Orkney Islands in 1903 [22]. Records of Antarctic Lithodidae date back to an individual of *Paralomis birsteini* collected by the RV Eltanin in 1967 from near Scott Island, north of the Ross Sea [23] and they were not recorded again until 1994 near Peter I Island [24]. There are a total of nine encounters with Lithodidae recorded from the slope/shelf of Antarctica with the earliest dating back to 1998 [25]. García Raso et al. [26] suggested that many of the observed distribution patterns of Antarctic lithodids were an artefact of limited sampling rather than representing true distributions. Earlier studies on the biogeography of the SO concentrated on the decapods as a whole [27]–[28], and predate most records of crabs south of 60°S. Hall & Thatje [29] examined the link between the biogeography of the Lithodidae and seafloor temperature and were the first to quantify the known temperature limits of the SO lithodids, giving examples of cold tolerant and cold intolerant species from the three genera present in the region.

The first crabs to be labelled as ‘invasive’ in Antarctic waters were two individuals of *Hyas araneus* found at the South Shetland Islands [30]. Tavares and De Melo [30] postulated that these brachyuran crabs, usually found in the North Atlantic and Arctic Oceans, entered the Antarctic either on ships’ sea-chests or through ballast water. The first study to suggest that lithodids were potentially ‘recolonising’ or ‘invading’ the Antarctic shelf as result of climate change was Thatje et al. [14]. This was also the first study to suggest that adult lithodid crabs crossing the deep sea was the most likely route for any invasion, although others had suggested a longer scale recolonistation of the Antarctic through the deep sea by the eurybathic genus *Neolithodes* from regions further north [31]. As well as the ‘invasion hypothesis’, Thatje et al., [14] suggested a direct detrimental impact as a result of this invasion on the native benthic fauna of Antarctica. Apart from more descriptive and biogeographic works (e.g., [32]), almost every subsequent publication which includes records of SO lithodids mentions the ‘invasion hypothesis’ [15], [29], [33]–[37]. Perhaps the strongest proponent of the lithodid ‘invasion’ hypothesis to date was a study by Aronson et al. [33], which stated that the Lithodidae may have been absent from Antarctic shelf waters for more than 14 million years (Myr).

The first publication to provide evidence for the lithodid ‘invasion’ hypothesis was a study by Smith et al. [16]. Based on a single ROV dive at depths of >850 m in the Palmer Deep (West Antarctic Peninsula), this paper set a minimum temperature tolerance of 1.4°C for the lithodid crab *Neolithodes yaldwyni*. It also predicted: 1) a ‘potential invasion’ of the surrounding shallower waters within one or two decades if current rates of warming in the area continued and 2) a detrimental impact upon the surrounding fauna by predation and also through changes to the local physical environment by the mixing of the surface sediments by the crabs [16]. Here, we assess in detail evidence for this ‘invasion’ of the Antarctic shelf by present day lithodid crabs by providing the first detailed examination of their modern and fossil record.

## Materials and Methods

In this study, we examined both fossil records and a database of Recent crabs and lobsters from the Southern Hemisphere.

Fossil data was compiled from all available published sources and from the British Antarctic survey fossil collection in Cambridge (Appendix S1). The Antarctic fossil decapod record covers the Jurassic to the Pliocene (approximately 150 My) but lacks data for many time periods (Fig. 2). The dataset includes 31 genera from 23 families representing seven infraorders (Astacidea, Glypheidea, Axiidea, Achelata, Polychelida, Anomura and Brachyura) of decapod crustaceans.

A database of 16,210 records of Recent crabs and lobsters from the Southern Hemisphere was compiled from the published literature, validated records from the Ocean Biogeographic Information System (OBIS) and the Global Biodiversity Information Facility (GBIF) (both accessed December 2012) and from unpublished fisheries observer reports (Appendix S2). Only records of adult or juvenile animals were included, thus excluding the three known records of crab and lobster larval stages from the region [38]–[39]. The database included records for 37 species of Decapoda (4 nephropid lobsters, 1 munopsid, 1 *Kiwa*, 3 Brachyura and 32 species of Lithodidae). The Lithodidae included 4 genera; 9 species of *Lithodes*, 6 species of *Neolithodes*, 1 *Paralithodes* and 16 *Paralomis*. Taxonomy for both Recent and fossil records follows the classification given by De Grave *et al.* [40]. A GIS (Geographic Information System) was then used to map and analyse the data and to overlay it on environmental data (bathymetry, oceanographic fronts and seafloor temperature) (Fig. 1). Seafloor temperature data was produced as described in Clarke *et al.* [41]. PRIMER 6 software [42] was used to analyse the geographic relationships amongst lithodid species in the Southern Hemisphere and discern biogeographic patterns within the Lithodidae. Faunal similarity between regions was quantitatively measured using Bray-Curtis similarities of presence/absence data.

The term Southern Ocean (SO) is used when referring to the Antarctic and the sub-Antarctic islands, outside, but close to and influenced by, the PF, e.g. the Prince Edward Islands, Crozet Islands and Kerguelen Islands [43]. Antarctic waters are defined as being those south of 60°S [29]. The Antarctic shelf break was defined as 1,000 m [44]. Samples were defined as coming from the Antarctic slope/shelf by using a GIS to select for those <50 km north of the 1,000 m bathymetric contour of the Antarctic continent.

## Results

### Fossil Record

The available decapod fossil record reveals large gaps in knowledge during part of the Cretaceous due to a lack of suitable marine deposits in Antarctica. The Late Cretaceous through to the end of the Eocene shows the highest diversity at family level and most fossil records across all infraorders (Fig. 2). Although there are marine fossil sites for the Oligocene through to the Pleistocene they are generally made up of drill holes or short stratigraphic sequences. Only two families from the decapod fossil record exist south of 60°S at the present day, the Nephropidae and the Galatheidae. Both are known from single living records found in deep water. There is no Antarctic fossil record for the most abundant family of Recent crabs, the Lithodidae (Fig. 2).

### Recent Geographic Distribution

To date 22 species of crabs and lobsters are recorded from the SO (Table 1). The known distributions of all Recent Lithodidae in the SO are constrained by temperature with no records from areas where water temperatures are lower than 0°C (Fig. 1).

**Table 1:** Classification and distribution of Recent Southern Ocean crabs and lobsters. Classification taken from De Grave*et al.,* 2009.

Infraorder	Family	Species	Range	Depth (m)
ANOMURA	Kiwaidae	*Kiwa* sp.	Endemic to hydrothermal vents on East Scotia Ridge	2400–2600
	Lithodidae	*Lithodes macquariae* *Ahyong, 2010*	Macquarie Island and Solander Trough	16–1140
		*Lithodes murrayi*Henderson, 1888	4 records within the PF from Peter I Is., Records from Crozet,Amsterdam & St Paul, Reunion and Prince Edward Islands.(Records from NZ, Macquarie and South Africa were revisedsee Ahyong, 2010)	90–1030
		*Neolithodes capensis*Benedict, 1895	South Africa also recorded from the Kerguelen Plateau andBellingshausen Sea	660–3200
		*Neolithodes diomedeae* *(Benedict, 1895)*	Shag Rocks/South Georgia with records from southern SouthAmerica, the Eastern Pacific and a single record from Peter I Is	602–2454
		*Neolithodes duhameli*Macpherson, 2004	Only recorded at Crozet Is	1040–1500
		*Neolithodes yaldwyni* *Ahyong, 2010*	Endemic to waters south of 60° mainly in Ross Sea butalso Amundsen and Bellingshausen Seas	170–1950
		*Paralomis aculeata*Henderson, 1888	Only recorded from Prince Edward Islands, Crozetand Ob Bank	360–1500
		*Paralomis anamerae*Macpherson, 1988	Only found inside the PF (Shag Rocks/South Georgia and Ob & Lena Banks)	262–1997
		*Paralomis birsteini*Macpherson, 1988	Has a Ross Sea/Antarctic Peninsula centred distributionwith sub-Ant records from Crozet, Kerguelen,Tasmania & Macquarie	456–2003
		*Paralomis elongata*Spiridonov *et al.,* 2006	Only recorded at Bouvet Island	300–320
		*Paralomis formosa*Henderson, 1888	From the tip of South American shelf to Shag Rocks/South Georgiaand the South Sandwich Islands	145–2125
		*Paralomis spinosissima*Birstein & Vinogradov, 1972	From the tip of South American shelf to ShagRocks/South Georgia	121–1690
		*Paralomis stevensi*Ahyong & Dawson, 2006	Endemic to waters south of 60° mainly in Ross Sea butalso Amundsen Sea	1327–1924
	Galatheidae	*Munidopsis albatrossae*Pequegnat & Pequegnat,1973	Eastern Pacific and 1 record from Bellingshausen Sea	1920–3680
BRACHYURA	Hymenosomatidae	*Halicarcinus planatus*(Fabricius, 1775)	Southern South America, Marion Is., Kerguelen Is. and 1record from Macquarie Is. and 1 record from theSouth Orkney Is	0–126
	Oregoniidae	*Hyas araneus*(Linnaeus, 1758)	North Atlantic and 2 individuals recorded fromthe South Shetland Is in 1986 (not recordedagain since)	0–402
	Epialtidae	*Rochinia gracilipes*Milne-Edwards, 1875	Coast of Brazil and 1 individual recorded from theSouth Shetland Is (not recorded again since)	18–58
ASTACIDEA	Nephropidae	*Thymopides grobovi*(Burukovsky & Averin, 1976)	Only found on the Kerguelen Plateau	708–1010
		*Thymops birsteini* (Zarenkov& Semenov, 1972)	South America and Shag Rocks/South Georgia	394–1666
		*Thymops takedai*Ahyong *et al.,* 2012	South America and Shag Rocks/South Georgia	265–1739
		*Thymopsis nilenta*Holthuis, 1974	Scotia Sea (Shag Rocks/South Georgia &South Sandwich Islands)	822–3040

**Figure 3:**
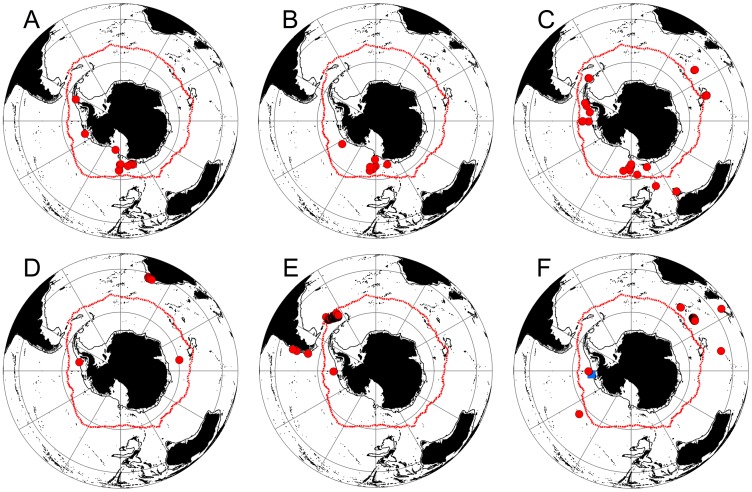
Distributions of anomuran crab species found south 60°S. Neolithodes yaldwyni (A), Paralomis stevensi (B), Paralomis birsteini (C), Neolithodes capensis (D), Neolithodes diomedeae (E), Lithodes murrayi (F – red circle) and Munidopsis albatrossae (F – blue square). Red line = Mean position of Polar Front (Moore et al., 1999).

**Figure 4:**
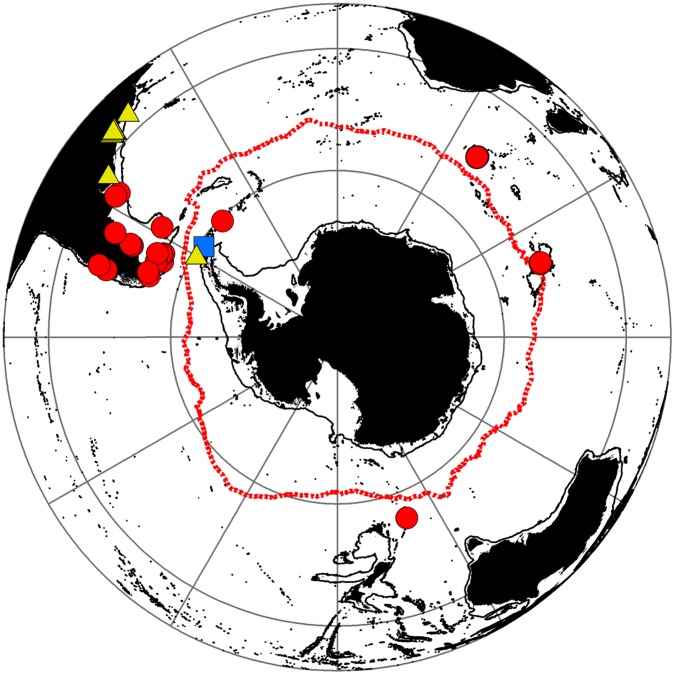
Distribution of brachyuran crabs found south of 60°S. Red circle = *Halicarcinus planatus* = , Blue square = *Hyas araneus* and Yellow triangle = *Rochinia gracilipes*. Red line = Mean position of Polar Front (Moore et al., 1999).

**Figure 5:**
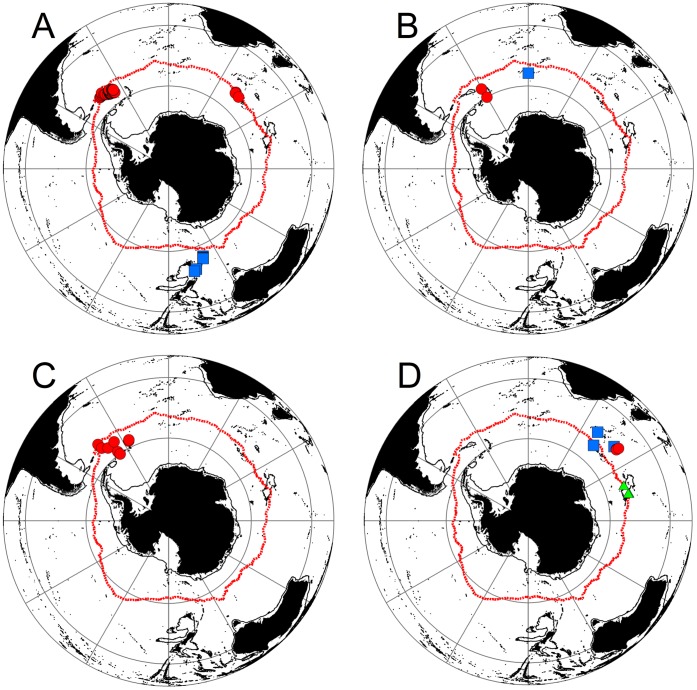
Southern Ocean crabs and lobsters with distributions south of the sub-Antarctic Front. *Paralomis anamerae* (A – Red circle), *Lithodes macquariae* (A – Blue square), *Kiwa sp*. (B – Red circle), *Paralomis elongata* (B – Blue square), *Thymopsis nilenta* (C), *Thymopides grobovi* (D – Green triangle), *Paralomis aculeata* (D – Blue squares) and *Neolithodes duhameli* (D – Red circle). Red line = Mean position of Polar Front (Moore et al., 1999).

There are six species of Lithodidae found south of 60°S (Fig. 3), representing 137 animals from 61 separate locations. Two species of lithodids have only ever been found south of 60°S, N. *yaldwyni* and *P. stevensi*. A single record of *M. albatrossae* exists from the slope of the Antarctic Peninsula in the Bellingshausen Sea (Fig. 3). The Brachyura are represented by three species south of 60°S. A single record of *H. planatus* is known from the South Orkney Islands [22]. Most records of this species come from South America and the sub-Antarctic (Fig. 4). Two further species of Brachyura have been recorded from the South Shetland Islands, *R. gracilipes* (usually found in South American waters) and *H. araneus* (a North Atlantic species) [45], [30] (Fig. 4). None of these brachyuran species have been collected in the region since. Other species with records south of 60°S are an undescribed *Kiwa* sp. and the lobster *T. nilenta,* but samples were taken from the Scotia Arc just south of 60°S, well away from the continent itself (Fig. 5).

There are nine recorded encounters with lithodids (observations or specimens captured) on or near to the Antarctic shelf/slope (<50 km from the 1000 m bathymetric contour). These nine encounters represent 62 individuals from four species (44 *N. yaldwyni*, 16 *P. birsteini*, 1 *N. capensis* and 1 *P. stevensi*) (Fig. 3). These records range from 850 m to 1947 m deep and occur from the slope of the Ross Sea through to the North West end of the Antarctic Peninsula (64.9°S–75.5°S & 178.7°W - 64.3°W). No records of Lithodids exist for the Antarctic shelf/slope beyond the Ross, Amundsen and Bellingshausen Seas.

**Figure 6:**
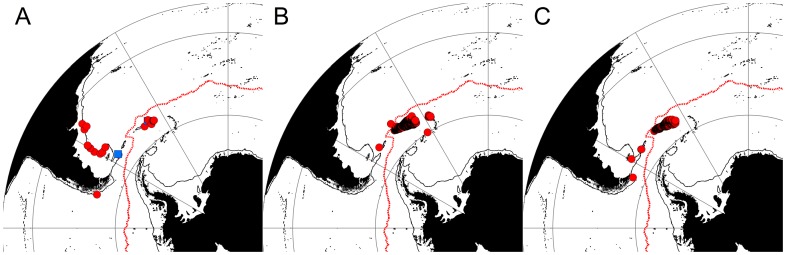
Crab and lobster species shared only between Southern South America and the northern Scotia arc. *Thymops takedai* (A – Blue Square), *Thymops birsteini* (A – Red circle), *Paralomis formosa* (B) and *Paralomis spinosissima* (C). Red line = Mean position of Polar Front (Moore et al., 1999).

Other SO species restricted to south of the sub-Antarctic Front or near to the PF include five species of Lithodidae (*L. macquariae*, *P. anamerae, P. elongata, P. aculeata* and *N. duhameli*), one species of Kiwaidae and two species of Nephropidae (*T. nilenta* and *T. grobovi*) (Fig. 5). A further two species of lobster, *T. takedai* and *T. birsteini,* and two lithodids, *P. formosa* and *P. spinosissima* have been found to span the Drake Passage with records in southern South America and South Georgia (Fig. 6).

### Regional Diversity

Different geographic regions displayed differing numbers of taxa and different species compositions. In addition to the Antarctic slope/shelf (already discussed), regional diversity within the PF included three lithodid species from Peter I Island (*L. murrayi, N. diomedeae* and *P. birsteini*), three lithodid species from the Balleny Islands and neighbouring seamounts (*N. yaldwyni, P. birsteini* and *P. stevensi*) and five species from the Scotia arc (the hydrothermal vent *Kiwa* sp., the brachyuran *H. planatus,* the nephropid *T. nilenta* and two species of lithodid, *P. birsteini* and *P. formosa*). Spiess seamount near Bouvet Island has a single, endemic species of lithodid, *P. elongata*. The best sampled and most speciose region within the PF is the South Georgia/Shag Rocks region. This region has three species of Nephropidae (*T. birsteini, T. nilenta* and the recently described *T. takedai*) and four species of Lithodidae (*N. diomedeae, P. anamerae, P. formosa* and *P. spinosissima*). The sub-Antarctic islands and plateaus of the Southern Indian Ocean are home to a single, endemic, species of nephropid (*T. grobovi*), one species of brachyuran (*H. planatus*) and six species of lithodid (*L. murrayi, N. capensis, N. duhameli, P. aculeata, P. anamerae* and *P. birsteini*). Macquarie Island waters have records for *H. planatus, L. macquariae* and *P. birsteini*.

### Depth Distribution

**Figure 7:**
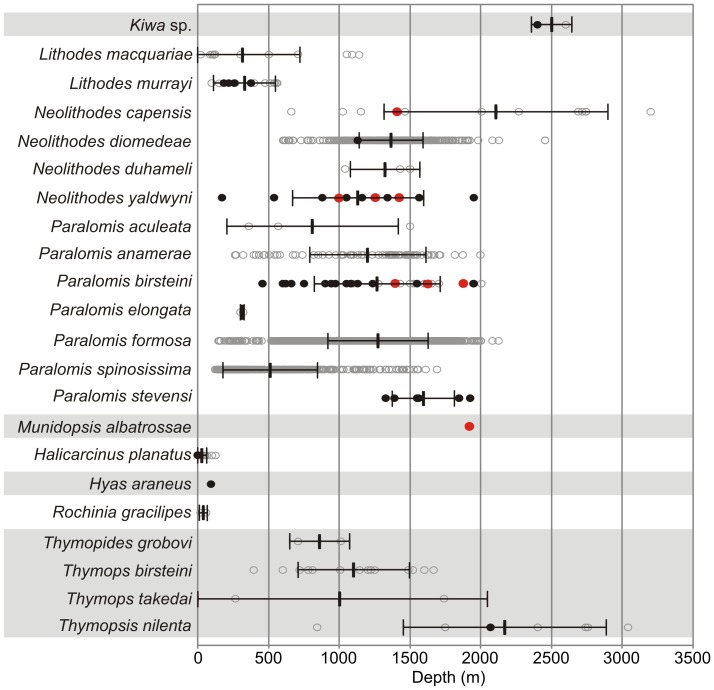
Depth ranges of Southern Ocean crabs and lobsters. Thick black vertical bars = mean depth of records, horizontal bars indicate standard deviation from that mean. Grey circles = all records from the Southern Ocean and neighbouring regions, black dots = records from south of 60°S, red dots = records from the Antarctic shelf/slope.

The majority of species examined in this study (13 out of 21) can be considered to be highly eurybathic, with depth ranges exceeding 1,000 m (Fig. 7). None of the Brachyura displayed depth ranges greater than ∼400 m. The species with the smallest depth ranges included those with only very few records e.g. newly discovered *Kiwa* sp., known from two locations in the Scotia Sea [46], and *P. elongata,* which is also recorded from two locations near Bouvet Island. In general, the nephropid lobsters displayed wide depth ranges e.g. *T. nilenta* with a depth range of 2,218 m. The lithodid genus *Paralomis* had the greatest number of species (5 species) with depth ranges wider than 1,000 m. The widest depth range examined belonged to the lithodid *N. capensis* with a depth range of 2,540 m (660–3,200 m).

The depths of samples taken from Antarctic waters (south of 60°S) and from the slope/shelf of the Antarctic continent tended to sit within the normal depth range of the species (Fig. 7). All Antarctic slope/shelf records, other than a single record for *P. birsteini* (1,947 m), are within the standard deviation of each species’ depth range. The only known record of *P. stevensi* from the Antarctic slope/shelf is lacking a depth record at its location (Ahyong, 2010), but bathymetric data suggest an approximate depth of 1,000 m, making it the shallowest known record for this species. The single record of *M. albatrossae* is the only record of this species in the SO and is its shallowest record to date (1,920 m).

The deepest records from Antarctic waters belonged to the *Kiwa* sp. (2,400 m), with *T. nilenta* second deepest at 2,068 m. All other Antarctic records are from depths shallower than 2,000 m. The brachyurans found south of 60°S have all been found shallower than 100 m.

### Biogeographic Patterns

**Figure 8:**
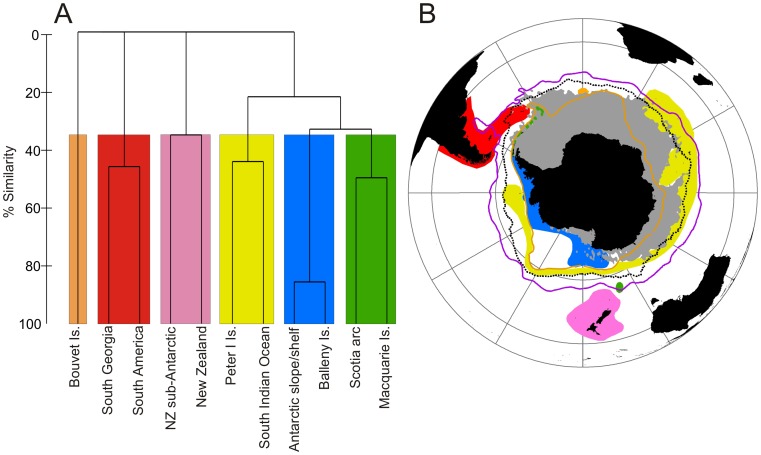
Biogeographic patterns in the distributions of the Lithodidae. Dendrogram of faunal similarity for Southern Ocean regions from Bray-Curtis similarities using complete linkage clustering. Coloured boxes represent biogeographic groupings at a 35% faunal similarity cut off value. Map of the geographic locations of the clusters. Black dotted line = Polar Front, purple solid line = Sub-Antarctic Front, orange solid line = southern Antarctic Circumpolar Current boundary (SACCB), grey = Seafloor temperature <0°C.

Since the Lithodidae had far more records available for analysis and a wider latitudinal range that the other taxa, we performed a biogeographic analysis of only this family. Six distinct geographic groupings were evident using a cut-off point of 35% similarity (Fig. 8a). These groupings were then mapped with seafloor temperature and the mean positions of oceanographic fronts for comparison (Fig. 8b). The most southerly grouping (blue) was made up of the continental margin regions south of the southern Antarctic Circumpolar Current boundary (SACCB), the West Antarctic slope/shelf and the Balleny Islands. The waters north of the SACCB but south of the sub-Antarctic Front included four of the biogeographic groupings. The smallest grouping was that of the Bouvet Island region, which is comprised of a single geographic region with a single endemic species. The widest longitudinal range of any grouping is the southern Indian Ocean Islands, plateaus and Peter I Island group. The third grouping within the Antarctic Circumpolar Current (ACC) was that of Macquarie Island and the Scotia arc. The fourth grouping to cross these frontal boundaries is the only one which connects Antarctic waters to more temperate ones (South Georgia and South America). The fifth grouping is comprised of New Zealand and the sub-Antarctic Islands of New Zealand, including 13 species not recorded in any of the other regions.

## Discussion

### Fossil Record

There are many controls on the preservation of decapods which make interpreting their evolutionary history difficult. A large percentage (99.7%) of the Antarctic continent is covered by snow and ice [47], restricting the exposure area in which fossils can be found, and resulting in an intermittent record. Most decapods come from geological units that span long time intervals. These are normally the sites which have the best exposures, i.e. the largest exposed surface (e.g., Seymour Island). Deep Sea Drilling Program (DSDP) drill holes have a long time range but no decapod fossil record (Fig. 2), most likely because exposure is restricted to the drill width, but also because fossil decapods are rare in Neogene Antarctica fossil sites [48].

Preservation (taphonomy) also needs to be considered when interpreting the decapod fossil record. Fossil arthropods in the record are largely a result of exceptional preservation conditions [49], and a substantial loss of arthropods has been noted during the preservation of communities [50]–[51]. Experimental taphonomy has shown that crabs have a high susceptibility to decay early in the post mortem history [52]. This may explain the lack of evidence, in Antarctica and globally, for fossil lithodids. As taphonomic controls on the preservation of decapods are so important, changes in the depositional environment could be a major controlling factor on the presence of decapods in the fossil record. The greatest diversity in Antarctic fossil crabs is seen in sites, such as those on Seymour Island, of shallow and medium depth water (Fig. 2).

The La Meseta Formation on Seymour Island is the most diverse unit for fossil decapods [53], and an important site for investigating possible relationships between cooling temperatures and an associated decline in predation pressure from taxa such as decapods. Dense monospecific assemblages of ophiuroids and crinoids in the upper few meters of the formation show little evidence for predation [54]–[55]. Aronson and Blake [56] stated that the position of the echinoderm assemblages puts them ‘close to the end of the La Meseta time when crabs and sharks had essentially disappeared and teleosts may well have been in decline’. This has been used to support the theory that the Late Eocene/Early Oligocene cooling was the cause of the decrease and disappearance of decapods in Antarctica [55].

However, Feldmann *et al.* [57] note that decapod diversity is actually greatest in the lower and upper parts of the La Meseta Formation. Despite the observed decrease in sub lethal predation, decapod diversity still remained high in the final unit of the formation (Feldmann *et al*. [57]). Therefore, the suggested reduction and elimination of decapods in Antarctica, which is proposed to have originated with cooling in the Eocene to Oligocene [33], [55], may not have occurred until a much later time period, when cooling was even greater. Decapods are recorded from two other localities at a time when temperature was lower than the Eocene to Early Oligocene. Decapods form a dominant part of the Early Miocene Cape Melville Formation (CMF) community [17]. There is also a record of a lobster from the Pliocene.

When discussing the fossil history of the SO decapods, the fossil record of the La Meseta Formation on Seymour Island is often regarded as documenting the decline (and in some papers, the extinction) of large decapods in Antarctica [33], [54]–[56]. This fossil record is composed entirely of the Brachyura and Nephropidae, and does not include any Lithodidae. We consider that La Meseta Formation should not be regarded as an analogue for the anomuran Lithodidae because they display very different Recent distribution patterns to the modern day Brachyura [28].

In summary, virtually nothing is known of the history of Lithodidae from the fossil record (Fig. 2), although some conclusions on the global origins of the group have been drawn through molecular methods and Recent depth distribution patterns [36].

### Ice Sheet History and Palaeoceanography

Although the fossil record is of little use for comparing past and present day distributions of anomuran Lithodidae, large areas of ice free and relatively undisturbed slope and shelf have been available for colonisation around Antarctica and the sub-Antarctic Islands as ice sheets retreated after the Last Glacial Maximum (LGM; c. 18,000–20,000 years ago).

On the western Antarctic Peninsula, shelf habitats similar to the present day could have existed in northern areas since the onset of post-LGM glacial retreat, c.18,000-17,000 years ago [58]–[62]. Further south, the Marguerite Bay ice stream retreated rapidly from the outer shelf region from about 14,000 years ago, possibly associated with rapid sea level rise during Meltwater Pulse 1-a (MWP 1-a), before stabilising, and then retreating further from the mid-shelf sometime prior to c. 10,500-9,500 years ago [62]–[66]. The inner-shelf area has been deglaciated since at least 9,500-7,500 years ago [58], [61]–[62] when the ice stream thinned rapidly and isostatic rebound caused relative sea levels to fall rapidly in the Marguerite Bay area from c. 41 m above present, sometime after c. 9,000 years ago, to c. 19 m above present by c. 7,000 years ago [67].

Intrusion of relatively warm (>1°C) Circumpolar Deep Water (CDW) flooding onto the continental shelf, up to a depth of 200 m, has been suggested as a possible casual mechanism for the final phase of ice retreat in Marguerite Bay during the Early Holocene [61]–[62], [65]. Intrusion of CDW has also been linked to:

1. Southwards retreat of the George VI Ice Shelf from Marguerite Bay to at least the Ablation Point area between 9,600-7,500 years ago [68]–[70].
2. Deglaciation of the inner shelf area of the Bellingshausen Sea, which occurred as early as c. 14,000 years ago [71].
3. Present day and past retreat of the Pine Island Glacier in the Amundsen Sea [72].

In the western Amundsen Sea region, deglaciation from the outer shelf could have begun as early as c. 22,000 years ago [73], likely reaching the mid-shelf area through a series of ‘episodic’ phases of grounding line retreat between 16,400-12,200 years ago, which have also been associated with the rapidly rising sea levels of MWP 1-a [72]. Ice from the western and eastern Amundsen sea areas coalesced into a single Amundsen Sea Ice-Shelf, which briefly stabilised on the inner shelf area 12,200-10,600 years ago, before rapidly collapsing sometime after 10,600 years ago. Deglaciation from the outer to inner Pine Island Bay area was driven initially by intrusion of CDW 7,800-7,000 years ago, with further retreat during the last 7000 years caused by increased input of subglacial water to the bottom of the ice shelf [72].

Periodic intrusion of CDW onto some parts the West Antarctic shelf has probably occurred since the Early Holocene, but is likely to have occurred during ‘warmer’ phases of the Mid-Late Holocene. For example, Bentley *et al.* [74] suggested that southwards shifts in the Southern Hemisphere Westerlies could have led to an intensification of the ACC during the Mid Holocene Hypsithermal (4500-2800 years ago), which was sufficient to drive CDW onto the western shelf of the Antarctic Peninsula.

In summary, a significant and increasingly larger area of the outer to inner shelf from the tip of the Antarctic Peninsula to the Amundsen Sea has become available for colonisation over the last c. 20,000 years. Shelf areas have remained largely ice-free and relatively undisturbed by ice near, or shortly after, the end of the Early Holocene climate optimum 9,500 years ago, and likely experienced episodic intrusions of CDW since, which could have lasted for significant periods of time.

In comparison, the extent and timing of glacier expansion on South Georgia at the LGM remains largely unknown because none of the moraines on the inner and outer shelf have been numerically dated. Decreased moisture availability associated with the advance of sea ice limits and changes in the intensity of, and/or a latitudinal shift in, the Southern Westerlies could have resulted in a relatively limited expansion of LGM ice. However, geomorphological evidence exists for a more extensive ice sheet characterised by ice streams flowing from the central highlands through fjords, which are connected to large cross-shelf troughs [75]–[82]. Onshore and terrestrial data suggests that present day shelf habitats in northern coastal areas around Cumberland Bay became ice-free c. 11,000 years ago, with a few protected areas located within glacial troughs becoming ice-free as early as c. 17,000 years ago [83]. Significant disturbance of outer and inner shelf habitats is, therefore, unlikely during the last c. 11,000 years because glacier activity on the northern coast has been restricted to fjords and inland coastal and mountain areas since the Early Holocene climate optimum (11,000-9500 years ago) [74], [76], [78], [80], [83]–[86].

### Recent Sampling Effort

Over 99% of the 16,210 records compiled for this study were for the Lithodidae including the vast majority of Antarctic records (53 of a total of 59 sites south of 60°S). All non-lithodid taxa were represented at single sampling locations south of 60°S, but varied in numbers of individuals from a single animal (*M. albatrossae, H. planatus* and *R. gracilipes*), a pair of *H. araneus* to >700 individuals m^−2^ of the *Kiwa* sp. [87]. The vast majority of SO crab and lobster records (∼70%) come from deeper than 1,000 m, including all records from the Antarctic slope/shelf. This is almost the exact opposite of records for most other groups of benthic organisms in the region. In total, 70% of benthic sampling stations from the SO are from shallower than 1,000 m deep [10]. South of 60°S, 92% of benthic samples are from <1,000 m deep, with 78% of Ross Sea benthic invertebrate samples and 76% of West Antarctic Peninsula samples from shallower than 600 m (SOMBASE data for benthic isopods, molluscs, crabs and pycnogonids). These two regions account for a large proportion of all recorded benthic sampling in the Antarctic, 18% from the WAP and 22% from the Ross Sea region. Prior to the 1970s less than 1% of all known benthic sampling from south of 60°S was deeper than 900 m. Since 1970s approximately 8% of Antarctic sampling has been from deeper than 900 m and of this 36% has taken place in the Weddell Sea. No crabs or lobsters have ever been recorded from the Weddell Sea. This low number of encounters on or around Antarctica can be explained by decreasing densities of animals with depth [88] accompanied by a far smaller number of samples taken from deeper water [12].

Analysis of the literature that reports crab catches, scientific research vessels have reported 162 crab catches out of 971 deployments of gear in waters south of the PF ([16], [20], [24]–[26], [32], [34]–[35], [89]–[91], BIOPEARL II). Of these, 97 were Agassiz trawls with a crab catch rate of 10 hauls (∼10%), 68 of 375 baited traps or cameras (17%), 79 of 422 beam or bottom trawls (∼19%) and 4 out of 27 ROV transects (∼15%). It is important to note that these samples are reported from publications which included records of crabs or lobsters, but the majority of sampling in the SO does not include records of crabs.

Many samples are taken with grabs, box corers or sledges which are unlikely to catch crabs. García Raso *et al.* [35] report a single munopsid in 25 Agassiz trawl deployments, but none using a supra-benthic sled deployed in the same locations. Unfortunately, no database currently exists for every deployment, gear and subsequent catches in the SO, so it remains impossible to assess the true effectiveness of each gear type. It is likely that there is considerable variability in the efficacy of each gear type used to sample lithodids. Large commercial-sized bottom trawls and baited traps and cameras are likely to be far more effective at assessing crab presence than small Agassiz type trawls or non-baited cameras and ROVs. As a result it is difficult to compare relative abundance between regions and depths where both the intensity of sampling and the range of gear used has been highly variable.

Even around South Georgia, where considerable directed and non-directed sampling effort for crabs has been carried out for over two decades [37] there remain areas for which information on lithodid distribution remains limited. Scientific observations show that lithodid crabs are rare if not absent in the very shallow waters (<100 m) [92]. Regular demersal fish surveys sample depths between 100–350 m whilst exploratory pot fisheries for crabs have operated at depths of <1000 m, however, depths between 400 m and 1,000 m remain relatively poorly sampled [37]. Lithodid distribution records therefore must be considered in light of the degree and type of sampling carried out in a particular location.

### Recent Geographic Distributions

Seafloor water temperature appears to be a major determining factor in the geographic distribution of lithodid crabs in Antarctica (Fig. 1) as distributions are likely affected by physiological constraints. Hall & Thatje [29] presented evidence for a sharp temperature cut-off point for most species of SO lithodids of around 0.5°C with 4 species (*N. yaldwyni, P. stevensi, P. formosa and P. birsteini)* having ranges extending to between 0.5°C and ∼0°C. Data analysed in this study (Fig. 1) support this, and, in addition, show that no records of lithodids exist in areas colder than 0°C, even in the very well sampled areas of shelf such as the Eastern Weddell Sea or the shelf of the Ross Sea. Smith *et al.,* [16] give a lower temperature tolerance limit of 1.4°C for *N. yaldwyni* from their observations in the Palmer Deep basin, strongly contradicting Hall & Thatje [29] and this study, where 1.4°C is at the higher end of temperatures recorded for this species. Smith *et al.,* [16] cite their findings as being an important model for potential invasive species because the Palmer Deep seafloor water temperatures have risen from 1.20°C to 1.47°C since 1982. Smith *et al.,* [16] state that this temperature rise enabled *N. yaldwyni* to colonise the region for the first time around 1998 (when temperature reached 1.4°C). The temperature ranges for *N. yaldwyni* observed by Hall & Thatje [29] and this study strongly suggest that the temperatures recorded in the Palmer Deep region since 1982 have not been sufficiently cold to exclude the species as they have always been at least 1°C higher than their lower temperature tolerance limit. The most likely explanation for the presence of *N. yaldwyni* in the Palmer Deep is, as postulated by Smith *et al*. [16], pulsed larval transport from the continental slope across the shelf with the regular incursions of warm CDW, but not necessarily over the timescales suggested.

The distribution of *L. murrayi* in Antarctic waters is restricted to the seafloor around Peter I Island (Fig. 3f), most likely Peter I Island is located in an oceanographically favourable position, in the currents of the ACC, north of SACCB. The ACC is rich in warm CDW [93], and Klages et al. [24] recorded in situ temperatures of 1.8°C at the seafloor locations where the *L. murrayi* were observed. The other two species recorded from Peter I Island, *N. diomedeae* and *P. birsteini*, are only found at greater depths (621–1129 m) where temperatures are cold, but remain >1°C down to depths exceeding 1,500 m [24]. This southerly limit for *L. murrayi* could represent its thermal tolerance limit as its range does not extend onto the Antarctic shelf or slope. No regions of the seafloor of the West Antarctic Peninsula reach temperatures of 1.8°C [41]. Although Hall and Thatje [29] give a lower temperature limit for *L. murrayi* of ∼1°C, this possibly reflects the one degree precision of gridding in the Southern Ocean Atlas [94], which would not be able to adequately resolve small area represented by the Peter I Island shelf at the warmer temperatures.

The SO crabs and lobsters examined show some typical distribution patterns similar to those observed in other benthic species such as the Pycnogonida and Porifera [95]–[96]. The main difference in the distribution of these decapods is that they are not found in the cold (<0°C) regions of the continental margin. The presence of two endemic species, *N. yaldwyni* and *P. stevensi*, and an Antarctic centred distribution for *P. birsteini* implies a regionally distinct fauna and localised speciation.

### Recent Regional Diversity

Given that the Antarctic shelf/slope has only had nine recorded lithodid encounters, dating back to 1998, it is somewhat surprising that it shows a higher diversity of lithodid species than the better sampled Scotia arc (2 species), Balleny Islands (3 species) and Peter I Island (3 species) and equalling the diversity of the extensively sampled South Georgia (4 species from 3,325 records). This relatively high diversity from a low number of samples, including two endemic species, suggests a community that has had more than a few centuries or millennia to develop.

### Recent Depth Distributions

Other than *L. murrayi* and the Brachyura, all other species found south of 60°S showed predominantly deep water distributions and the Antarctic shelf/slope specimens were all from depths of around 1000 m or deeper. These distributions are relatively normal for lithodids outside of the Northern Pacific [36], with very few Southern Hemisphere species having shallow water records. The deepest records of most Southern Hemisphere lithodids do not extend beyond ∼2,500 m [36], however, this study found records of *N. capensis* at depths of 3,200 m off the coast of South Africa. This deep water preference of the lithodids, especially those from beyond the Northern Pacific (Hall & Thatje, 2009) implies that these species are unlikely to rapidly spread into shallower water over a short timescale.

Excluding *L. murrayi* (discussed previously), the Lithodids with normal depth distributions which extended into shelf depths (<500 m) were all north of 60°S, *L. macquariae*, *P. aculeata, P. elongata* and *P. spinosissima*. The vast majority of records from south of 60°S are deeper than 500 m, only two individuals of *N. yaldwyni* and *P. birsteini* have been found shallower than this depth.

The absence of shallower dwelling species from shelf areas south of 60°S [29] (other than at the unusually warm Peter I Island), contrasts sharply with their presence on the sub-Antarctic slope and shelf environments. In the sub-Antarctic and around South Georgia, a limited expansion of independent ice caps during the LGM might not have covered the entire shelf area, allowing shallower water species to persist. In contrast LGM ice covered most of the Antarctic shelf, likely eradicating any shallow dwelling Antarctic lithodids and suitable habitats. Nevertheless, shallower habitats along the entire West Antarctic shelf have been deglaciated for at least 9,000 years, with periodic intrusion of CDW onto the shelf area likely providing them with habitable (>0°C) conditions. The Antarctic lithodids have had sufficient time to colonise the deglaciated West Antarctic shelf, but have not done so, even when oceanographic conditions during some ‘warmer’ phases of the last 9000 years (e.g., Mid Holocene Hypsithermal (MHH) 4500-2800 years ago; [74]) are thought to have been similar, in some areas, to those of the present day [74]. Instead, the Antarctic lithodids have remained in the deeper water (>1,000 m) of the slope or shelf basins (Fig. 7).

### Recent Biogeographic Patterns

Previous studies [27]–[28] considered the Decapoda as a whole and did not distinguish between benthic and pelagic taxa in their biogeographic classifications. The study by Hall & Thatje [29] compared bathymetric, geographic and temperature ranges for SO lithodids, but did not define any biogeographic regions from their analyses.

The biogeography of the SO lithodids appears to be driven by a combination of seafloor temperature and oceanographic fronts. Geographic isolation and a largely deep sea fauna have been used in the past to explain the high levels of endemism at Bouvet [43]. These factors, coupled with its apparent thermal isolation (Figs 1 & 8), with colder waters to the South and West, have probably restricted the endemic species, *Paralomis elongata*, to the region and prevented further colonisation by other species. The frontal systems of the ACC appear to delineate the major biogeographic groupings of the Antarctic and sub-Antarctic. The SACCB represents the northern boundary for the strongest grouping, the Antarctic slope/shelf and Balleny Islands (86% similarity), which is further restricted longitudinally by the sub-zero waters of East Antarctica and the Weddell Sea. Conversely, the SACCB represents the southern boundary of the grouping spanning from the Southern Indian Ocean to Peter I Island. For this group of animals, unlike many other benthic taxa [43], [94]–[95], the PF does not seem to represent a substantial biogeographic boundary. Once again contrasting with other studied taxa [43], [94], the lithodids of South Georgia group more closely with those of southern South America than they do with the Antarctic. The main feature which connects South America and South Georgia and could provide a walkable route into the SO is the North Scotia Ridge, a submarine feature which runs between the tip of South America and Shag Rocks at depths shallower than 2,000 m. The grouping of the Scotia Arc and Macquarie Island can be explained by the low lithodid diversity in both regions, two recorded species per region, with both including the circumpolar species *P. birsteini* (Fig. 3c). As with other benthic taxa, the lithodid fauna of New Zealand and its sub-Antarctic Islands does not strongly group with those of the SO [43], [94]–[95], probably reflecting the long term isolation of New Zealand and its wide latitudinal and habitat ranges encompassing sub-Antarctic to sub-tropical habitats [43].

### Invasion Hypothesis

The World Conservation Union (IUCN) define invasive species as “an alien species which becomes established in natural or semi-natural ecosystems or habitat, is an agent of change, and threatens native biological diversity”. Many authors in the past have used the term invasive interchangeably with range extension or polar emergence. It is important to differentiate between introduced, non-native species and native taxas which are expanding or changing their bathymetric or geographic ranges due to the effect of climate change. Other than in the case of *Hyas araneus* [30], most authors who describe potential “invasion” of the high Antarctic shelf by lithodids are in fact referring to potential range extensions or the theory of polar emergence [14]–[15], [29], [33]–[37].

Recent studies have suggested a deep sea migration of adult crabs into Antarctic waters from regions further north [14], [16]. The average depth of the seafloor south of the PF is ∼3,700 m and 88% of it is deeper than 2,000 m, with 56% of SO seafloor being colder than 0°C [12], [41]. The oceanographic nature of the biogeographic groupings coupled with all Antarctic lithodid records coming from shallower than 2,000 m implies that dispersal by adult crabs walking across the abyssal seafloor at the present day may be restricted. This would suggest that the ACC could also play some role in dispersal of juveniles or larvae.

If the presence of lithodids in the Antarctic were a simple case of recolonisation, then diversity levels and species composition would be expected to reflect this [97]–[98] with a generally lower diversity and reflecting a sub-set of the fauna further north. The Recent Antarctic lithodid distribution patterns do not fit this pattern (Figs. 1 & 8). The present day distribution pattern, including 2 endemic species (Fig. 1), of lithodids around Antarctica imply a long and enduring existence in the region. This suggests an evolutionary history including a continued lithodid presence either on or near to the Antarctic slope during times of expanded ice sheets.

The presence of only deep-water species of lithodids and the absence of any shallow water species, e.g. the South American *Lithodes santolla* (depth range from intertidal to >200 m, mean depth of 80 m from over 11,000 records) support the idea of a community which had a deep water refuge. The exact nature of the oceanography and seafloor temperatures of the West Antarctic slope during the LGM remains unknown (as does the presence or absence of cold bottom water production along this continental margin due to a lack of suitable data from south of the PF). Nevertheless, the geographic and depth distribution of warm CDW off the West Antarctic during the LGM is thought to have been broadly similar to the present day [99], but with CDW upwelling located further from the continental margin, especially in regions of ABW production [100]. This may have allowed the current deep water lithodids to have existed in situ, even through the coldest parts of the last ice age, most likely on the seamounts and islands in the region. Balleny and Peter I Islands, and the Marie Byrd and De Gerlache Seamounts and the seamounts to the west of the Antarctic Peninsula, are likely to have remained as suitable habitats (or refugia) for lithodid crabs in West Antarctica.

### Effect on the Native Fauna

A number of recent studies [14]–[16] have suggested that the potential colonisation of Antarctic shallow marine ecosystems by ‘durophagous’ lithodid crabs poses a considerable threat to benthic invertebrate communities in these regions. It has been speculated that weakly calcified invertebrates such as molluscs would be at risk of predation by ‘bone crushing’ (durophagous) lithodid ‘predators’. However, there is currently very little information available on the diet and feeding behaviour of the deepwater lithodids that are found south of the PF. There is strong evidence that many of the deepwater lithodids in the region are opportunistic necrophagous scavengers [90] and are attracted to foodfalls and carrion. Very high densities of *P. spinosissima*, *P. formosa* and *N. diomedeae* are known to be attracted to dead squid bait. The diet of *N. diomedeae* has also been shown to consist largely of deep-sea organic debris of pelagic origin [101]. The shallower living *L. murrayi* has a very diverse diet and is considered an opportunistic forager which also includes a high degree of necrophagy [102]. The diets of lithodids such as the Pacific King Crab, *Paralithodes camtschatica* (Tilesius, 1815) from the northern hemisphere are much better studied e.g. [103] and are known to feed on bivalves, gastropods and echinoderms. However these groups are known to be less well represented in the diet of *L. murrayi* [102]. It therefore remains uncertain as to whether there would be an increased risk of predation to sedentary invertebrate organisms in the high Antarctic shelf sea regions if lithodids were to colonise these habitats.

South Georgia is the largest island south of the PF and it is considered to be the most studied and speciose region of the SO recorded to date (1,445 species from over 17,000 digitised records [104]). It has a benthos which is generally Antarctic in character whilst also being rich in endemic species and high in numbers of range edge species [43], [104]–[105]. These include many of species of cnidarians (78 species), echinoderms (119) and molluscs (161) despite the presence of these large durophagous predators in high abundances. This highly diverse community exists and thrives in the presence of four species of lithodid crabs [37] and three species of Nephropidae. Over 3,000 records of lithodids from depths between 121 m and 2,100 m come from the South Georgia shelf. Lithodids are so widespread, and are found at such high densities in South Georgia that they have been the subject of repeated, but largely unsuccessful, exploratory fisheries since the early 1990s [106]–[107]. In the case of South Georgia, it appears that lithodid crabs can form part of a highly diverse community without detrimentally affecting species numbers or habitat type. Elsewhere, studies of the documented invasion of a non-native species of lithodid, *Paralithodes camtschaticus*, in the shallow waters of the Barents Sea found that the detrimental effect on the native benthos was not as dramatic as had been expected [108]. Despite the high feeding activity of *P. camtschaticus*, its omnivory is thought to have distributed the predation pressure across a range of taxa, thus preventing the elimination of any particular species [108].

### The Future of Antarctic Crab Populations

Significant oceanographic changes in the Antarctic have been confirmed through long term observations and repeated sampling. Surface waters around the West Antarctic Peninsula are known to have warmed significantly in recent decades, with a 1°C warming of the upper 25 m of the water column [4], as have seafloor temperatures in some areas, but to a lesser degree [16].

Although this study found little evidence for ongoing range expansion or polar emergence, these future scenarios cannot be ruled out given the current changes to the high Antarctic shelf habitats. Given that the SO Lithodidae show a preference for deeper water/slope habitats, one potential route for a range expansion would be longitudinally around the continental shelf margin into the Weddell Sea or East Antarctica. At the present day the sub-zero ABW (mostly formed in the Weddell and Ross Seas) acts as a biogeographic barrier to the lithodids [29]. However, ABW production is known to be changing around the continent including freshening and warming on decadal scales [109]–[113]. If significant long term changes to ABW temperature and distributions were to occur then the lithodids could potentially expand their longitudinal ranges. The likelihood and timescale of any such changes are still uncertain and these massive oceanographic and temperature changes would be far more detrimental to the Antarctic benthic ecosystem than the addition of a few crab species [5]–[8].

Studies on other Antarctic and sub-Antarctic groups, e.g. octopus [114]–[115], gastropods [116] and kelp [117], have shown long and complex histories linked to climatic and oceanographic events, which are reflected in their genetic patterns. These studies show how and, perhaps more importantly, when different taxa entered and left Antarctic waters, diversified, became isolated and speciated. Although some molecular work on lithodids has been carried out at the genus level on global lithodids by Hall and Thatje [36], and others have used DNA barcoding to identify Antarctic species [16], [34], there is a real lack of data suitable for population genetics studies. Until multiple individuals from multiple species at a variety of localities have been analysed for multi-gene loci the precise history and phylogeographic relationships of the Antarctic Lithodidae will remain a hotly debated topic.

### Conclusions

The relatively recent records of lithodid crabs living on the slopes of West Antarctica, the Ross Sea and in the overdeepend basins on the shelf, has led many to suggest that they are a new and expanding element of these marine communities. However, virtually no scientific work using well-established suitable sampling methods has been conducted in these regions below 1,000 m and, to date, no campaign has repeatedly targeted decapod distributions over time in a single geographic region to assess any density or distributional changes. Furthermore, the global fossil history of the deep dwelling Lithodidae is virtually non-existent and has often been confused with the shallower water fossil record of the Brachyura.

Using a database of 16,210 records of Recent crabs and lobsters from the Southern Hemisphere, compiled from the published literature using validated records from the Ocean Biogeographic Information System (OBIS) and the Global Biodiversity Information Facility (GBIF), we have found no evidence of any present day expansion in geographic or bathymetric range for any lithodid species in the SO. Future research in this area must clearly differentiate between describing potential non-native invasive species versus native species extending their range due to environmental change.

Given the sparse number of deep water samples taken, the known Antarctic lithodid fauna is relatively rich in species and includes taxa not found elsewhere. This endemicity is reflected in the biogeographic regions, which show a distinct assemblage south of the SACCB. These patterns also imply that lithodids have existed around Antarctica for long enough to have speciated and remained in situ.

Potential future changes to the production and temperature of ABW may facilitate an expansion of the ranges of lithodids into the Weddell Sea or East Antarctica. However, such fundamental oceanographic changes are likely to have a far greater effect on the native fauna than the effects caused by a change in crab distribution.

An integrated research program of repeated, hypothesis testing sampling of lithodid distributions in Antarctic waters is recommended to test the validity of recently published crab invasion hypotheses.

## Supporting Information

Appendix S1 **Data sources for Antarctic fossil crabs and lobsters used to create** **Figure 2** **.** Fossil data were compiled from all available published sources and from the British Antarctic survey fossil collection.
(DOC)

Appendix S2 Data sources for Recent crabs and lobsters from the Southern Hemisphere. Recent data were compiled from the published literature, validated online records and from unpublished fisheries observer reports.
(DOC)
