# Supplementary material for: Antarctic Crabs: Invasion or Endurance?
Source: PLoS One. 2013 Jul 3;8(7):e66981. doi: 10.1371/journal.pone.0066981 (PMC3700924; doi:10.1371/journal.pone.0066981)
Supplement: Appendix S1 — Data sources for Antarctic fossil crabs and lobsters used to create Figure 2 . Fossil data were compiled from all available published sources and from the British Antarctic survey fossil collection. (DOC) [file pone.0066981.s001.doc]

FOSSIL REFERENCES

**Classifications follow:**

De Grave S, Pentcheff ND, Ahyong ST, Chan TY, Crandall KA, et al. (2009) A classification of living and fossil genera of decapod crustaceans. Raffles B Zool 21: 1-109.

**For decapod Ranges:**

Aguirre-Urreta MB, Buatois LA, Chernoglasov GCHB. Medina FA (1990) First Polychelidae (Crustacea, Plinura) from the Jurassic of Antarctica. Antarct Sci 2: 157-162.

Aguirre-Urreta MB, Marenssi S, Santillana S (1995) A new Eocene crab (Crustacea, Decapoda) from Seymour Island, Antarctica. Antarct Sci 7: 277-281.

Ball HW (1960) Upper Cretaceous Decapoda and Serpulidae from James Ross Island, Graham Land. Falkland Islands Dependencies Survey Scientific Reports 24: 1-30.

Feldmann RM (1989) *Metanephrops jenkinsi* n. sp. (Decapoda: Nephropidae) from the Cretaceous and Paleocene of Seymour Island, Antarctica. J Paleontol 63: 64-69.

Feldmann RM (1992) The genus *Lyreidus* de Haan, 1839 (Crustacea, Decapoda, Raninidae): Systematics and biogeography. Antarctica. J Paleontol 66: 943-957.

Feldmann RM (1994) *Antarctomithrax thomsoni*, a new genus and species of crab (Brachyura; Majidae) from the La Meseta Formation (Eocene) of Seymour Island, Antarctica. J Paleontol 68: 174-176.

Feldmann RM,Wilson MT (1988) Eocene decapod crustaceans from Antarctica. Geo Soc Mem 169: 465-488.

Feldmann RM, Gażdzicki A (1997) A new species of *Glyphea* (Decapoda: Palinura) from the La Meseta Formation (Eocene) of Seymour Island, Antarctica. Acta Palaeontol Pol 42: 437-445.

Feldmann RM, Quilty PG (1997) First Pliocene decapod crustacean (Malacostraca: Palinuridae) from the Antarctic. Antarct Sci 9: 56-60.

Feldmann RM, Zinsmeister WJ (1984) New fossil crabs (Decapoda: Brachyura) from the La Meseta Formation (Eocene) of Antarctica: paleogeographic and biogeographic implications. J Paleontol 58: 1046-1061.

Feldmann RM, Zinsmeister WJ (1984) First occurrence of fossil decapod crustaceans (Callianassidae) from the McMurdo Sound region, Antarctica. J Paleontol 58: 1041-1045.

Feldmann RM, Crame JA (1998) The significance of a new nephropid lobster from the Miocene of Antarctica. Palaeontology 41: 807-814.

Feldmann RM, Schweitzer CE (2006) Paleobiogeography of Southern Hemisphere decapod Crustacea. J Paleontol 80: 83-103.

Feldmann RM, Tshudy DM, Thomson MRA (1993) Late Cretaceous and Paleocene Decapod Crustaceans from James Ross Basin, Antarctic Peninsula. Memoir of the Paleontological Society: 28, Supplement to J Paleontol 67: 1-41.

Feldmann RM, Schweitzer CE, Marenssi SA (2003) Decapod crustaceans from the Eocene La Meseta Formation, Seymour Island, Antarctica: a model for preservation of decapods. J Geol Soc 160: 151-160.

Förster R (1985) In: Förster R, Gaździcki A, Wrona R (1985) First record of a homolodromiid carb from a Lower Miocene glacio-marine sequence of West Antarctica. Neues Jahrb Geol P-M 6: 340-348.

Förster R, Gaździcki A, Wrona R (1987) Homolodromiid crabs from the Cape Melville Formation (Lower Miocene) of King George Island, West Antarctica. Acta Palaeontol Pol 49: 147-161.

Schweitzer CE and Feldmann RM 2000*. Callichirus? Symmetricus* (Decapoda:Thalassinoidea) and associated burrows, Eocene, Antarctica. *In* Paleobiology and Paleoenvironments of Eocene rocks, McMurdo Sound, East Antarctica. Antarct Res Ser 76: 335-347.

Taylor BJ (1979) Macrurous Decapoda from the Lower Cretaceous of South-Eastern Alexander Island. British Antarctic Survey Scientific Reports 81: 1-39.

Tshudy DM, Feldmann RM (1988) Macruran decapods, and their epibionts, from the López de Bertodano Formation (Upper Cretaceous), Seymour Island, Antarctica. Memoir of the Geological Society of America 169: 291-301.

Weller S (1903) The Stokes collection of Antarctic fossils. J Geol 11: 413-419.

**For Unit Dates:**

Ivancy LC, Lohmann KC, Hasiuk F, Blake DB, Glass A, Aronson RB, Moody RM (2008) Eocene climate record of a high southern latitude continental shelf: Seymour Island, Antarctica. Geoll Soc Am Bull 120: 659-678.

**For Water Depths:**

Feldmann RM, Wilson MT (1988) Eocene decapod crustaceans from Antarctica. Geo Soc Mem 169: 465-488.

Feldmann RM, Tshudy DM, Thomson MRA (1993) Late Cretaceous and Paleocene Decapod Crustaceans from James Ross Basin, Antarctic Peninsula. Memoir of the Paleontological Society: 28, Supplement to J Paleontol 67: 1-41.

Feldmann RM, Quilty PG (1997) First Pliocene decapod crustacean (Malacostraca: Palinuridae) from the Antarctic. Antarct Sci 9, 56-60.

Hara U, Crame JA (2004) A new aspidostomatid bryozoans from the Cape Melville Formation (Lower Miocene) of King George Island, West Antarctica. Antarct Sci 16, 319-327.

Taylor BJ (1979) Macrurous Decapoda from the Lower Cretaceous of South-Eastern Alexander Island. British Antarctic Survey Scientific Reports 81: 1-39.

Schweitzer CE, Feldmann RM (2000)*. Callichirus? symmetricus* (Decapoda:Thalassinoidea) and associated burrows, Eocene, Antarctica. In Paleobiology and Paleoenvironments of Eocene rocks, McMurdo Sound, East Antarctica. Antarct Res Ser 76: 335-347.

Smith AB, Crame JA (2012) Echinoderm faunas from the Lower Cretaceous (Aptian-Albian) of Alexander Island, Antarctica. Palaeontology 55: 305-324.

Stilwell JD, Zinsmeister WJ, Oleinik AE (2004) Early Paleocene Mollusks of Antarctica: Systematics, Paleoecology and Paleobiogeographic Significance. Bulletins of American Paleontology 367: 1-89.

Whitham AG (1993) Facies and depositional processes in an Upper Jurassic to Lower Cretaceous pelagic sedimentary sequence, Antarctica. Sedimentology 40: 331-349.

**Dates for fossil sites:**

Buckeridge JS (1989) Marine invertebrates from late Cainozoic deposits in the McMurdo Sound region, Antarctica. J Roy Soc New Zeal 19: 333-342.

Dingle RV, Lavelle M (1998) Antarctic Peninsular cryosphere: Early Oligocene (c. 30 Ma) initiation and a revised glacial chronology. J Geol Soc 155: 433-437.

Gaździcki A, Tatur A, Hara U, Del Valle RA (2004) The Weddell Sea Formation: post−Late Pliocene glacial deposits on Seymour Island, Antarctica. Pol Polar Res 25: 189-204.

Harwood DM, Levy RH (2000) The McMurdo erratics: Introduction and overview. In Paleobiology and Paleoenvironments of Eocene rocks, McMurdo Sound, East Antarctica. Antarct Res Ser 76: 1-18.

Ivany LC, Lohmann KC, Hasiuk F, Blake DB, Glass A, Aronson RB, Moody RM (2008) Eocene climate record of a high southern latitude continental shelf: Seymour Island, Antarctica. Geoll Soc Am Bull 120: 659-678.

# Jonkers HA, Kelley SP (**1998)** A reassessment of the age of the Cockburn Island Formation, northern Antarctic Peninsula, and its palaeoclimatic implications. **J Geoll Soc London 155: 737**-**740.**

Leckie RM,Webb PN (1983) Late Paleogene and Early Neogene foraminifers of Deep Sea Drilling Project Site 270, Ross Sea, Antarctica. *In*: Initial Reports of the Deep Sea Drilling Project VOLUME XC, Part 1 covering Leg 90 of the cruises of the Drilling Vessel *Glomar Challenger* Noumea, New Caledonia, to Wellington, New Zealand, December 1982-January 1983.

Prentice ML, Bockheim JG, Wilson SC, Burckle LH, Hodell DA, Schlüchter C, Kellogg DE (1993) Late Neogene Antarctic glacial history: Evidence from Central Wright Valley. The Antarctic Paleoenvironment: a Perspective on Global Change. Antarct Res Ser 60: 207-250.

Pirrie D, Crame JA, Lomas SA, Riding JB (1997) Late Cretaceous stratigraphy of the Admiralty Sound region, James Ross Basin, Antarctica. Cretaceous Res 18: 109-137.

Quilty PG, Lirio JM, Jillett D (2000) Stratigraphy of the Pliocene Sørsdal Formation, Marine Plain, Vestfold Hills, East Antarctica. Antarct Sci 12: 205-216.

Stilwell JD, Harwood DM, Whitehead JM (2002) Mid-Tertiary macroinvertebrate-rich clasts from the Battye Glacier Formation, Prince Charles Mountains, East Antarctica. Antarct Sci 14: 69-73.

Webb PN, Andreasen JE (1986) Potassium/argon dating of the volcanic material associated with the Pliocene Pecten Conglomerate (Cockburn Island) and Scallop Hill Formation (McMurdo Sound). Antarct J US 21: 59.
