# Supplementary material for: Antarctic Crabs: Invasion or Endurance?
Source: PLoS One. 2013 Jul 3;8(7):e66981. doi: 10.1371/journal.pone.0066981 (PMC3700924; doi:10.1371/journal.pone.0066981)
Supplement: Appendix S2 — Data sources for Recent crabs and lobsters from the Southern Hemisphere. Recent data were compiled from the published literature, validated online records and from unpublished fisheries observer reports. (DOC) [file pone.0066981.s002.doc]

| **RECENT REFERENCES:** |
| --- |
| Amaral ACZ, Rizzo AE, Arruda EP (Ed.) (2005) Manual de Identificacao dos Invertebrados Marinhos da Regiao Sudeste-Sul do Brasil Editora da Universidade de Sao Paulo (EDUSP) - Sao Paulo. 1-270. |
| Arana PM, Retamal MA (1999) Nueva distribución de *Paralomis birsteini* Macpherson 1988 en aguas antárticas (Anomura, Lithodidae, Lithodinae). Invest. Mar, Valparaíso 27: 101-110.  Ahyong ST (2010) King crabs of New Zealand, Australia and the Ross Sea (Crustacea: Decapoda: Lithodidae). NIWA Biodiv Mem 123:1-194. |
| Belchier M, Peatman T, Brown J, Street K C (2012) The biology, ecology and development of fishery management advice for the anomuran crabs at South Georgia (CCAMLR subarea 48.3). CCAMLR Sci 19: 1-15. |
| BIOPEARL I & II Expeditions (2006 & 2008) British Antarctic Survey (Unpublished data). |
| Collins MA, Yau C, Guilfoyle F, Bagley P, Everson I, Priede I G, Agnew D (2002) Assessment of stone crab (Lithodidae) density on the South Georgia slope using baited video cameras. ICES Journal of Marine Science: Journal du Conseil 59: 370-379. |
| De Melo GAS (1995) Manual de identificação dos Brachyura (caranguejos e siris) do litoral Brasileiro. São Paulo: Plêiade/FAPESP 603 pp. |
| Department of Invertebrate Zoology, Research and Collections Information System, NMNH, Smithsonian Institution. See: http://www.mnh.si.edu/rc/db/2data_access_policy.html (accessed through GBIF data portal, <http://data.gbif.org/datasets/resource/1834> 2013-03-07). |
| García Raso JE, García Muñoz JE, Manjón-Cabeza ME (2008) First record of *Munidopsis albatrossae* (Crustacea: Decapoda: Galatheidae) from Antarctic waters. Polar Biol 31: 1281-1285. |
| García Raso JE, Manjón-Cabeza ME, Ramos A, Olasi I (2005) New record of Lithodidae (Crustacea, Decapoda, Anomura) from the Antarctic (Bellingshausen Sea). Polar Biol 28:642-646. |
| Gutt J, Helsen E, Arntz WE, Buschmann A (1999) Biodiversity and community structure of the mega-epibenthos in the Magellan region (South America). Scientia Marina 63: 155-170. |
| Hale HM, Johnston TH (1941) Decapod Crustacea. BANZAR Expedition Committee. |
| Henderson JR (1888) Report on the Anomura collected by HMS Challenger during the years 1873-76. Eyre & Spottiswoode. |
| Kensley B (1977) The South African Museum's Meiring Naude cruises. Part 2. Crustacea, Decapoda, Anomura and Brachyura. Annals of the South African Museum 72: 161-188. |
| Kensley B (1980) Decapod and isopod crustaceans from the west coast of southern Africa, including seamounts Vema and Tripp. Annals of the South African Museum 83. |
| Klages M, Gutt J, Starmans A, Bruns T (1995) Stone crabs close to the Antarctic continent: *Lithodes murrayi* Henderson, 1888 (Crustacea; Decapoda; Anomura) off Peter I Island (68°51 S, 91°51 W). Polar Biol 15:73-75. |
| Komatsu H, Okuno J, Fukuoka K (2012) Studies on Eumalacostraca: a homage to Masatsune Takeda (Vol. 17). Brill. |
| Ledoyer M (1979) *Thymopides grobovi* (Burukovski et averin, 1976)(Crustacea, Decapoda, Nephropidae) Récolté aux iles kerguelen au cours des campagnes du MS" Marion Dufresne" en 1974 et 1975. Tethys 9: 123-128. |
| López Abellan LJ, Balguerías E (1993) On the presence of *Paralomis spinosissima* and *Paralomis formosa* in catches taken during the Spanish Survey ANTARTIDA 8611. CCAMLR Sci 1:165-173. |
| Lovrich GA, Perroni M, Vinuesa JH, Tapella F, Chizzini A, Romero MC, (2002) Occurrence of *Lithodes confundens* (Decapoda: Anomura) in the intertidal of the southwestern Atlantic. J Crustacean Biol 22: 894-902. |
| Lovrich GA, Thatje S, Calcagno J, Anger K, (2007) Las centollas colonizan la Antartida. In Ciencia Hoy, vol. 17 pp. 22-33. Bueno Aires, Argentina. |
| Macpherson E (2004) A new species and new records of lithodid crabs (Crustacea: Decapoda: Lithodidae) from the Crozet and Kerguelen Islands area (Subantarctica). Polar Biol 27: 418-422. |
| Miquel JC, Arnaud PM, Do-Chi T (1985) Population structure and migration of the stone crab *Lithodes murrayi* in the Crozet Islands, Subantarctic Indian Ocean. Mar Biol 89: 263-269. |
| Poupin J (2010) Biodiversité de l’Indo-Pacifique tropical français. |
| Programa de Observadores a Bordo (POBCh) de la Secretaria de Pesca de la Provincia del Chubut, Argentina . Observer On board Program -Fisheries Secretariat of the Province of Chubut-Argentina (OOBPPCh). |
| Pshenichnov LK (1996) Potentially commercial invertebrates on Ob Bank: *Moroteuthis ingens* (Oegopsida) and *Paralomis aculeata* (Anomura) (Division 58.4.4). CCAMLR WG-FSA-96/15.  Purves MG, Agnew DJ, Moreno G, Daw T, Yau C, Pilling G (2003) Distribution, demography, and discard mortality of crabs caught as by-catch in an experimental pot fishery for toothfish (*Dissostichus eleginoides*) in the South Atlantic. Fish Bull 101: 874–888. |
| Rios C, Mutschke E, Morrison E (2003) Benthic sublitoral biodiversity in the Strait of Magellan, Chile. Rev Biol Mar Oceanog 38: 1-12. |
| Rogers AD, Tyler PA, Connelly DP, Copley JT, James R, et al.(2012) The discovery of new deep-sea hydrothermal vent communities in the Southern Ocean and implications for biogeography. PLoS Biol 10: e1001234. |
| Selected verified records from South Western Pacific Regional OBIS Data provider for the NIWA Marine Biodata Information System (nzobis.niwa.co.nz), OBIS (www.iobis.org), GBIF ([www.gbif.org](http://www.gbif.org/)) and South Georgia Marine Biodiversity Database (www.antarctica.ac.uk/sgmarbase/) including South Georgia fisheries observer data. |
| Smith CR, Grange LJ, Naudts DL, Huber B, Guidi L, Domack E. (2012) A large population of king crabs in Palmer Deep on the west Antarctic Peninsula shelf and potential invasive impacts. P Roy Soc B-Biol Sci 279: 1730: 1017-1026. |
| Spiridonov V, Türkay M, Arntz WE, Thatje S (2006) A new species of the genus *Paralomis* (Crustacea: Decapoda: Lithodidae) from the Spiess seamount near Bouvet Island (Southern Ocean), with notes on habitat and ecology. Polar Biol 29: 137-146. |
| Stebbing TR (1914) IX. Stalk-eyed Crustacea Malacostraca of the Scottish National Antarctic Expedition. T Roy Soc Edin 50: 253-307. |
| Takeda M, Hatanaka H (1984) Records of decapod crustaceans from the Southwestern Atlantic collected by the Japanese Fisheries Research trawlers. Bull Nat Sci Mus A Zool 10:7-24. |
| Tavares M, De Melo GA (2004) Discovery of the first known benthic invasive species in the Southern Ocean: the North Atlantic spider crab *Hyas araneus* found in the Antarctic Peninsula. Antarct Sci 16: 129-131. |
| Thatje S, Hall S, Hauton C, Held C, Tyler P (2008) Encounter of lithodid crab *Paralomis birsteini* on the continental slope off Antarctica, sampled by ROV. Polar Biol 31: 1143-1148. |
| Yau C, Collins MA, Bagley PM, Everson I, Priede IG (2002) Scavenging by megabenthos and demersal fish on the South Georgia slope. Antarct Sci 14: 16-24. |
